# A randomized trial of nefopam for postoperative pain management in obstructive sleep apnea patients undergoing uvulopalatopharyngoplasty

**DOI:** 10.1007/s00405-026-10472-w

**Published:** 2026-09-16

**Authors:** Saowapark Chumpathong, Peerachatra Mangmeesri, Sriwimon Panyamee, Wish Banhiran, Maythad Uataya

**Affiliations:** 1 Department of Anesthesiology, Faculty of Medicine Siriraj Hospital, Mahidol University, Bangkok, Thailand; 2 Department of Otorhinolaryngology, Faculty of Medicine Siriraj Hospital, Mahidol University, Bangkok, Thailand

**Keywords:** Multimodal analgesia, Nefopam, Obstructive sleep apnea, Postoperative pain, Uvulopalatopharyngoplasty

## Abstract

**Objective:**

Uvulopalatopharyngoplasty (UPPP) is a surgical treatment for obstructive sleep apnea that often results in significant postoperative pain. Given the risks associated with opioid use in patients with obstructive sleep apnea, multimodal analgesia is preferred. This study evaluated whether nefopam, a non-opioid analgesic, without respiratory depressive effects, could reduce morphine consumption and improve postoperative recovery in patients undergoing UPPP.

**Methods:**

This prospective, exploratory, randomized, triple-blinded trial enrolled patients scheduled for elective UPPP. Participants were randomly assigned to receive either nefopam or a placebo (normal saline). The nefopam group received a 20 mg intravenous loading dose over 1 h, followed by a continuous infusion of 80 mg over 24 h. Both groups received standard multimodal analgesia. The primary outcome was total morphine consumption within 24 h postoperatively. The secondary outcomes were postoperative pain scores, time to first oral intake, patient satisfaction, quality-of-life scores, and adverse events.

**Results:**

Fifty-one patients were analyzed (nefopam: 26, control: 25). The median 24-hour morphine consumption was 7 mg in the nefopam group and 5 mg in the control group (*P* = 0.56). No significant differences were found between groups in postoperative pain scores, time to first oral intake, patient satisfaction, or quality-of-life scores. Adverse event rates were similar, although tachycardia (11.5%) and sweating (3.8%) were observed only in the nefopam group. Subgroup analysis in UPPP alone and extended UPPP surgery likewise revealed no significant differences between groups.

**Conclusion:**

Nefopam is not recommended for pain management following UPPP as it did not significantly reduce morphine consumption or improve pain-related outcomes.

## Introduction

Uvulopalatopharyngoplasty (UPPP) is a surgical treatment for obstructive sleep apnea (OSA) and snoring. The procedure involves removing or repositioning tissue in the oropharynx to widen, open, or stiffen the upper airway. UPPP is typically considered for patients with anatomical abnormalities contributing to OSA and is indicated when nonsurgical treatments, such as positive airway pressure therapy, weight reduction in obese individuals, or oral appliances, have failed or been declined [1]. 

Although UPPP can improve airway patency, it is often associated with significant postoperative pain and discomfort, which may persist for up to 2 weeks and interfere with daily activities [2]. Opioids are commonly used for postoperative pain management; however, in OSA patients, opioid use carries a heightened risk of respiratory depression due to increased tissue swelling following surgery. As a result, multimodal analgesia is preferred to minimize opioid-related complications. Despite the routine use of intravenous nonsteroidal anti-inflammatory drugs (NSAIDs) and local anesthetic administration, many patients still experience severe postoperative pain.

Nefopam, a nonopioid, non-NSAID analgesic, has gained attention as a potential alternative for postoperative pain management. Unlike opioids, nefopam does not cause respiratory depression, making it a suitable option for OSA patients. Previous studies have demonstrated its efficacy in various surgical settings, including hip replacement, laparoscopic cholecystectomy, and upper abdominal surgeries.

This study evaluated whether nefopam reduces morphine consumption in the first 24 h after UPPP. Additionally, we assessed its effects on postoperative pain, quality of life, and patient satisfaction.

## Methods

### Study design and setting

This prospective, randomized, triple-blinded trial was conducted at a university-based hospital in Bangkok, Thailand, between October 2021 and February 2024. The study was approved by the Siriraj Institutional Review Board (Si 170/2021) and registered with the Thai Clinical Trials Registry (TCTR20210203007).

### Patient selection

Patients undergoing elective UPPP were eligible for inclusion. The procedure involved the partial resection of the uvula, soft palate, and tonsils, particularly in cases of tonsillar hypertrophy, recurrent tonsillar infections, or tonsillolithiasis. Surgery was performed using a dissector and monopolar and bipolar cautery, followed by reconstruction of the palatopharyngeal tissues with absorbable sutures. Some patients also underwent radiofrequency ablation for inferior turbinate or base of tongue reduction.

Eligible participants were adults aged 18 to 65 years with a body mass index of less than 40 kg/m². They had to be capable of self-administering pain relief using intravenous patient-controlled analgesia and able to communicate in Thai. Patients were excluded if they had allergies or contraindications to study medications, including nefopam, morphine, lidocaine, parecoxib, etoricoxib, paracetamol, or dexamethasone. Additional exclusion criteria included concurrent use of opioids, monoamine oxidase inhibitors, selective serotonin reuptake inhibitors, or other pain relievers, as well as impaired liver function classified as Child‒Pugh B or C, creatinine clearance below 60 ml/min, or a history of glaucoma or epilepsy.

### Randomization and blinding

Participants were randomly assigned in a 1:1 ratio to either the nefopam or control group using a computerized block randomization method (block size of four). The control group received normal saline. To maintain blinding, the research staff prepared and labeled the treatment drugs as “research drugs,” ensuring that patients, anesthesiologists, surgeons, and data collectors remained unaware of group allocation. Informed consent was obtained before enrollment, and all participants completed the Thai version of the EQ-5D-5 L quality-of-life questionnaire [3] at baseline. This validated instrument assesses five dimensions of health—mobility, self-care, usual activities, pain/discomfort, and depression—on a scale from 0 to 1.

### Anesthesia and perioperative care

Two hours before surgery, all patients received paracetamol at a dose of 500 to 1000 mg, adjusted for body weight. General anesthesia was induced under standard monitoring using propofol (1‒2 mg/kg), fentanyl (1‒2 mcg/kg), and cisatracurium (0.1‒0.2 mg/kg). Anesthesia was maintained with desflurane, and intermittent fentanyl doses (0.5 mcg/kg) were administered every hour. After induction, dexamethasone (10 mg) and parecoxib (40 mg) were given. Before tissue resection, local infiltration with 6 to 8 mL of 1% lidocaine with adrenaline (1:200 000) was performed at the anterior pillars. At the conclusion of surgery, ondansetron (0.15 mg/kg) was administered for postoperative nausea prophylaxis. Muscle relaxation was reversed using atropine (20 mcg/kg) and neostigmine (50 mcg/kg). Patients were extubated once they met extubation criteria and were then transferred to the postanesthesia care unit (PACU).

### Intervention and control groups

Patients in the nefopam group received nefopam (Acupan, Delpharm Tours, France) as an intravenous infusion, with an initial dose of 20 mg in 100 mL of normal saline administered over 1 h at the start of surgery. This was followed by a continuous infusion of 80 mg of nefopam in 500 mL of normal saline at a rate of 20 mL per hour for 24 h. The control group received an equivalent volume of normal saline following the same administration protocol.

### Postoperative pain management

Postoperative pain was assessed using a numeric rating scale. In the PACU, patients with a pain score of 7 or higher received morphine at 2 mg every 5 min, while those with a score of 4 or higher received 1 mg every 5 min until their pain score dropped below 4. All patients were provided with an intravenous patient-controlled analgesia device programmed to deliver morphine at a dose of 1 mg, with a 5-minute lockout interval and a 4-hour maximum limit of 20 mg. No basal morphine infusion was used.

All patients received standardized multimodal analgesia. During the first 24 h postoperatively, this included paracetamol (1000 mg syrup) orally every 6 h, dexamethasone (10 mg intravenously) every 6 h, and parecoxib (40 mg intravenously) every 12 h. Patients were discharged on postoperative day 2. After discharge, between postoperative days 3 and 5, all patients were prescribed etoricoxib (90 mg once daily) and were permitted to take 1000 mg of syrup paracetamol as needed. This postoperative regimen was applied equally in both groups.

### Outcome measures and data collection

The primary outcome was total morphine consumption during the first 24 h postoperatively. Secondary outcomes included pain scores at rest and during swallowing over 21 days, the time to first morphine requirement, and the time to first oral intake of liquids and solid food. Adverse events, including tachycardia, nausea, vomiting, sweating, dizziness, urinary retention, confusion, and respiratory depression, were recorded. Patient satisfaction was assessed using a five-point scale (very satisfied, satisfied, neutral, unsatisfied, and very unsatisfied), and quality of life was measured using the EQ-5D-5 L questionnaire.

### Assessment timeline

Pain scores were recorded at multiple time points. In the PACU, assessments were performed at 30 and 60 min postoperatively. In the ward, nurses documented pain scores at 2, 12, and 24 h after surgery. Surgeons evaluated pain scores on postoperative days 1 and 2 and again before hospital discharge on day 2. Research assistants conducted follow-up assessments via telephone on postoperative days 3, 5, 7, 9, 14, and 21. Patient satisfaction and quality-of-life assessments were conducted on postoperative days 1, 7, and 14.

To maintain study integrity, all individuals involved in postoperative data collection, including patients, surgeons, PACU and ward nurses, and research assistants, were blinded to group allocations.

### Statistical analysis

We hypothesized that nefopam administration would result in a 30% reduction in postoperative morphine consumption. A previous study reported a median 24-hour morphine consumption of 86.7 mg following UPPP, with a standard deviation of 29 mg [4]. Using a two-sided type I error of 0.05 and 80% power, we estimated that a mean difference of 26 mg in 24-hour morphine consumption (86.7 mg vs. 60.7 mg), with a standard deviation of 29 mg, would require a sample size of 21 patients per group. To account for an anticipated 20% dropout rate, we increased the total sample size to 26 patients per group.

Descriptive statistics were used to summarize general patient characteristics. Quantitative variables were compared between groups using either a two-sample t test for normally distributed data or a Mann‒Whitney U test for nonnormally distributed data. Categorical variables were analyzed using Pearson’s chi-square test or Fisher’s exact test, as appropriate. A *P* value of less than 0.05 was considered statistically significant. All statistical analyses were performed using IBM SPSS version 29 (SPSS Inc, Chicago, IL, USA).

## Results

### Patient enrollment and study population

**Fig. 1:**
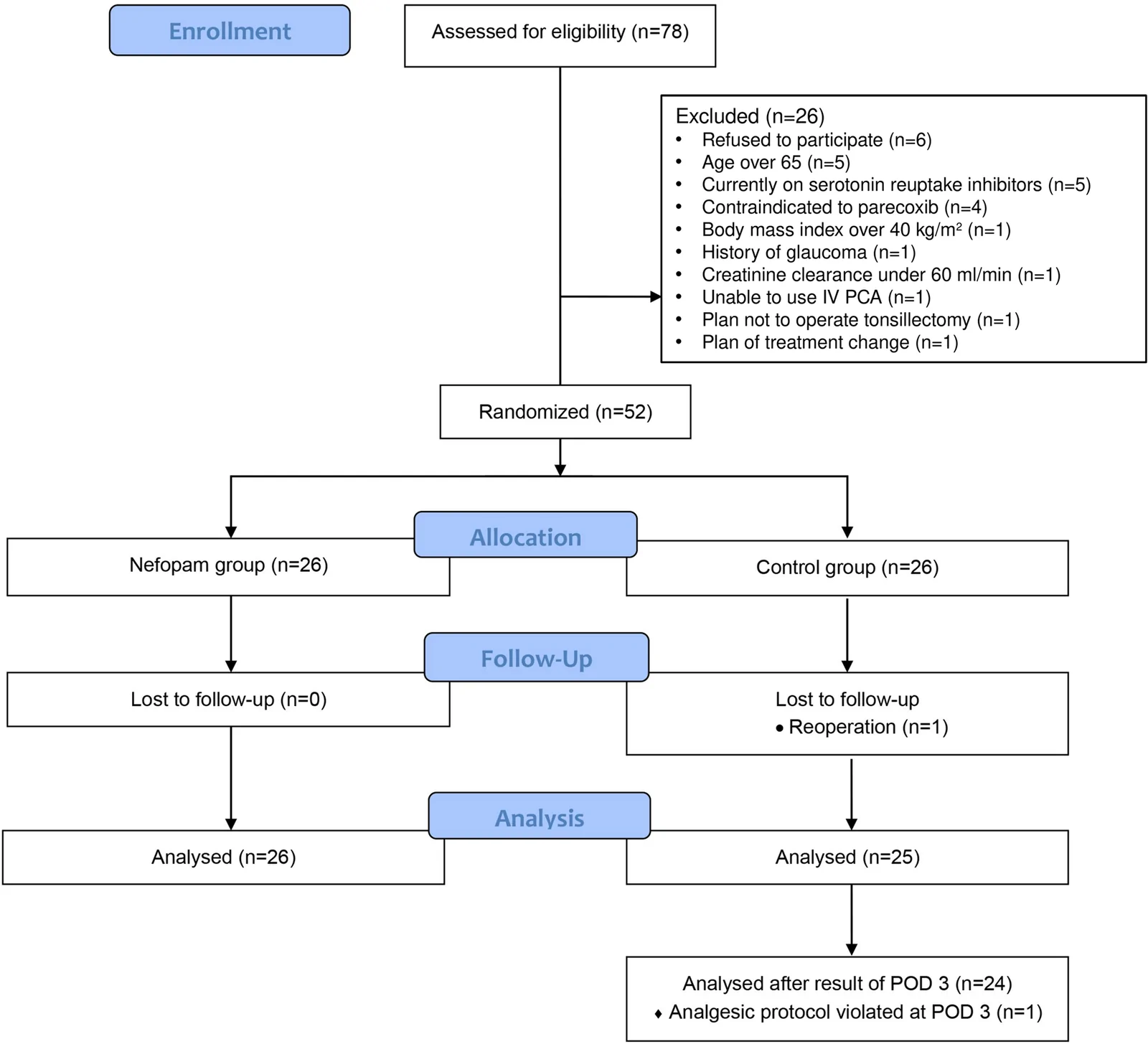
CONSORT diagram of the study. Abbreviations: IV PCA, intravenous patient-controlled analgesia; POD, postoperative day

Seventy-eight patients were screened for eligibility, of whom 52 were enrolled in the study. One participant in the control group was excluded due to postoperative bleeding that required reoperation at 8 h (Fig. 1). Consequently, 51 patients were included in the initial analysis. Additionally, one male patient (age 38) was partially analyzed up to postoperative day 3 before being excluded from further analysis due to protocol violation. This patient sought treatment at another hospital for severe pain and received an alternative analgesic regimen. Therefore, his data were included in the analysis up to 2 days postoperatively.

Baseline demographic and clinical characteristics were comparable between the two groups (Table 1).

**Table 1:** Patient characteristics by study group

Parameter	Control group (*n* = 25)	Nefopam group (*n* = 26)	*P* value
Sex: male	19 (76.0)	18 (69.2)	0.59
Age (years)	40.4 ± 10.9	39.0 ± 10.8	0.65
Body mass index (kg/m^2^)	27.9 ± 4.5	27.9 ± 4.2	0.99
ASA			
II	24 (96)	26 (100.0)	0.30
III	1 (4.0)	0 (0.0)	
Comorbidity			0.93
Diabetes	0 (0.0)	0 (0.0)	
Hypertension	8 (32.0)	8 (30.8)	
Quality-of-life score – baseline	1.0 (0.9, 1.0)	1.0 (0.8, 1.0)	0.23
Apnea-hypopnea index (/hour)			0.58
0–15	4 (18.2)	5 (19.2)	
15–30	4 (18.2)	8 (30.8)	
over 30	14 (63.6)	13 (50.0)	
Intraoperative fentanyl used (mcg/kg/hour)	1.2 ± 0.3	1.0 ± 0.3	0.23
Blood loss (ml)	20.0 (15.0, 50.0)	20.0 (10.0, 35.0)	0.10
Operation			0.28
UPPP	16 (64.0)	10 (38.5)	
UPPP and RF-IT	4 (16.0)	6 (23.1)	
UPPP and RF-BOT	3 (12.0)	4 (15.4)	
UPPP, RF-IT and RF-BOT	2 (8.0)	6 (23.1)	
Operative time (minutes)	107.2 ± 41.0	116.5 ± 35.4	0.39
Anesthetic time (minutes)	136.5 ± 36.6	146.7 ± 30.5	0.28
Hospital stay (days)	2.8 ± 0.7	2.6 ± 0.5	0.28

Values are presented as number (%), mean ± standard deviation (SD), or median (interquartile range)

*ASA* American Society of Anesthesiologists, *RF-BOT* radiofrequency for base of tongue reduction, *RF-IT* radiofrequency for inferior turbinate reduction, *UPPP* uvulopalatopharyngoplasty

### Postoperative morphine consumption and pain scores

**Fig. 2:**
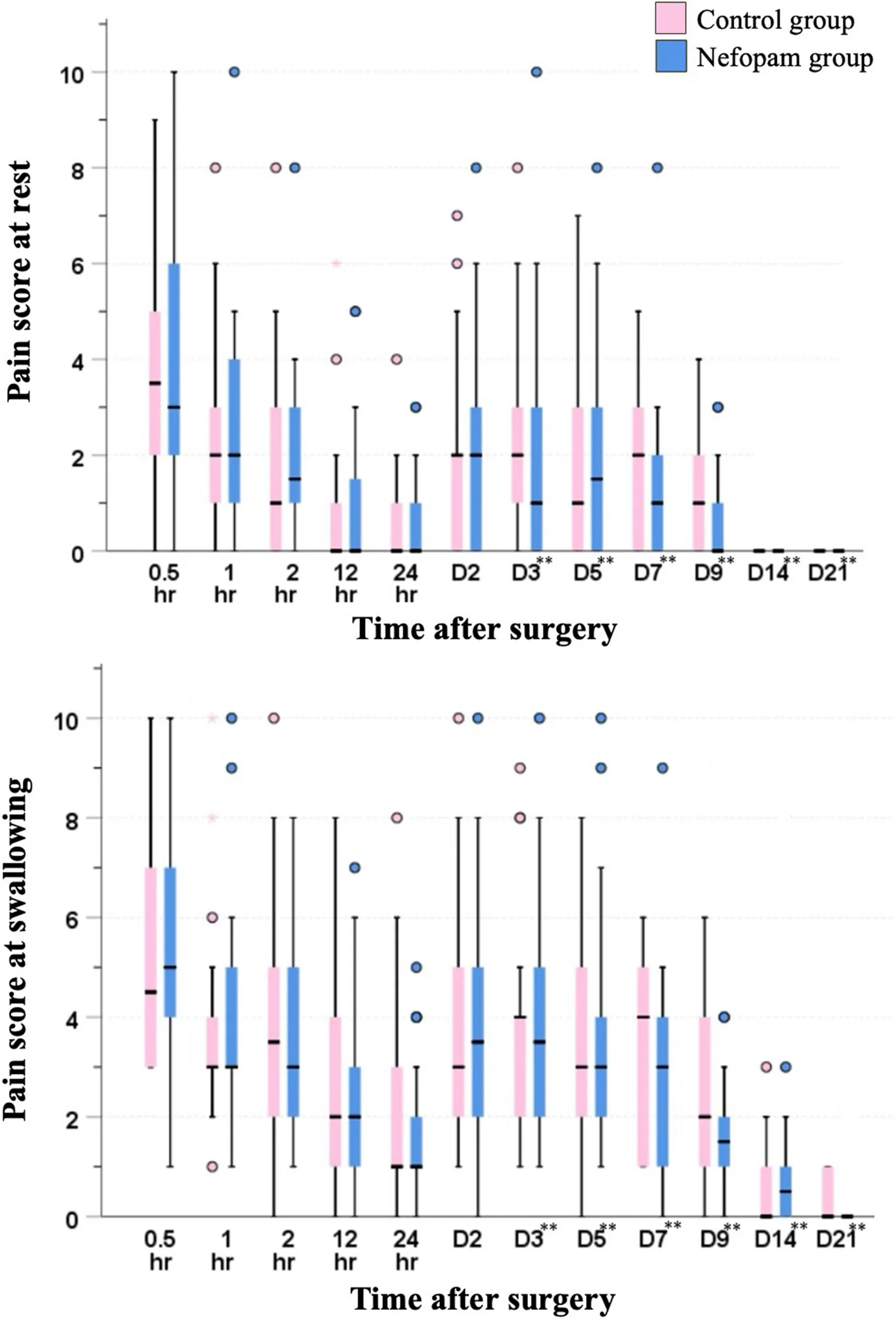
Comparison of median postoperative pain scores between the nefopam and control groups. ** The control group analysis was based on 24 patients due to one participant dropping out on postoperative day 3 following a research protocol deviation. Abbreviations: D, day; hr, hour

The median 24-hour morphine consumption was 7 mg in the nefopam group and 5 mg in the control group, with no statistically significant difference between the two (*P* = 0.56). Postoperative pain scores also showed no significant differences between the groups (Fig. 2). Similarly, the median time to first morphine dose, the time to first oral intake of liquids and solid food, and the quality-of-life scores did not differ significantly (Table 2). Patient satisfaction ratings at 24 h, 7 days, and 21 days postoperatively were also comparable between groups (Table 2).

**Table 2:** Postoperative recovery times, patient satisfaction, and quality-of-life scores

Parameter	Control group (*n* = 25)	Nefopam group (*n* = 26)	*P* value
Postoperative morphine consumption (mg)	5.0 (2.0, 12.0)	7.0 (3.2, 16.2)	0.56
Time of first morphine requirement in PACU (minutes)	10.0 (5.0, 20.0)	10.0 (7.5, 22.5)	0.66
Time until first drinking after surgery (minutes)	95.0 (71.5, 150.0)	75.0 (56.3, 141.3)	0.31
Time until first eating after surgery (minutes)	240.0 (187.5, 331.5)	287.5 (247.5, 364.0)	0.09
Patient satisfaction postoperative			
At 24 h			
Very satisfied	23 (92.0)	20 (76.9)	0.25
Satisfied	2 (8.0)	6 (23.1)	
At day 7*			
Very satisfied	15 (62.5)	16 (61.5)	1.00
Satisfied	8 (33.3)	8 (30.8)	
Neutral	1 (4.2)	2 (7.7)	
At day 14*			
Very satisfied	20 (83.3)	24 (92.3)	0.41
Satisfied	4 (16.7)	2 (7.7)	
Quality-of-life score			
At day 1	1.0 (0.5, 1.0)	1.0 (0.5, 1.0)	0.35
At day 7*	1.0 (0.9, 1.0)	1.0 (0.3, 1.0)	0.15
At day 14*	1.0 (1.0, 1.0)	1.0 (0.6, 1.0)	0.32

Values are presented as number (%), mean ± SD, or median (interquartile range)

*PACU* postanesthetic care unit

* The control group analysis at days 7 and 14 was based on 24 patients due to one participant dropping out on postoperative day 3 following a research protocol deviation.

### Pain progression and recovery

Patients in both groups reported greater pain during swallowing than at rest, particularly during the first postoperative week. While resting pain subsided within the first week, mild pain during swallowing persisted beyond this period. By the second postoperative week, most patients had returned to normal sensation with no residual pain. Despite experiencing pain in the first week, nearly all patients were able to resume basic daily activities by the time of hospital discharge, as assessed using the EQ-5D-5 L quality-of-life questionnaire.

### Subgroup analysis

We further analyzed outcomes according to the type of surgery, comparing patients who underwent UPPP alone with those who underwent UPPP combined with other procedures (radiofrequency for base of tongue reduction and/or inferior turbinate reduction). In both subgroups, there were no significant differences between the nefopam and control groups in median 24-hour morphine consumption, time to first morphine, time to first drinking, or time to first eating (Table 3).

**Table 3:** Subgroup analysis of nefopam versus control in patients undergoing UPPP alone or extended UPPP surgery

Parameter	UPPP only			Extended UPPP surgery*		
	Control (*n* = 16)	Nefopam (*n* = 10)	*P* value	Control (*n* = 9)	Nefopam (*n* = 16)	*P* value
Postoperative morphine consumption (mg)	7.0 (2.0, 12.8)	4.5 (2.5, 9.0)	0.54	4.0 (2.0, 11.5)	10.0 (3.3, 18.5)	0.29
Time of first morphine requirement in PACU (minutes)	10.0 (2.5, 20.0)	10.0 (10.0, 33.8)	0.18	10.0 (10.0, 20.0)	10.0 (5.0, 20.0)	0.35
Time until first drinking after surgery (minutes)	105.0 (69.8, 135.0)	105.0 (105.0, 172.5)	0.69	90.0 (67.5, 245.0)	65.0 (41.3, 123.8)	0.18
Time until first eating after surgery (minutes)	228.5 (183.8, 288.8)	290.0 (244.3, 374.0)	0.08	315.0 (187.5, 387.5)	287.5 (232.5, 362.5)	0.86

Values are presented as median (interquartile range)

*PACU* postanesthetic care unit, *UPPP* uvulopalatopharyngoplasty

* Extended UPPP surgery included UPPP and radiofrequency for base of tongue reduction and/or radiofrequency for inferior turbinate reduction

### Adverse events

The incidence of adverse events was similar between the two groups in both the PACU and the ward. In the PACU, nausea and vomiting occurred in one patient (3.8%) in the nefopam group, while dizziness was observed in three patients (12.0%) in the control group. In the ward, dizziness was reported in five patients (19.2%) in the nefopam group and three patients (12.0%) in the control group. Urinary retention was observed in two patients (7.7%) in the nefopam group and four patients (12.0%) in the control group.

In the nefopam group, tachycardia was reported in three patients (11.5%), and sweating was noted in one patient (3.8%); these adverse effects were not observed in the control group. No cases of respiratory depression or confusion were recorded in either group.

## Discussion

This randomized controlled trial evaluated the potential benefits of nefopam in patients undergoing UPPP. Our findings showed no significant reduction in 24-hour postoperative morphine consumption, pain scores, time to oral intake, or patient satisfaction in the nefopam group compared with the control group. The incidence of adverse events was also similar, although tachycardia and sweating were observed only in the nefopam group. Subgroup analyses likewise revealed no significant differences between groups.

UPPP is one of the most painful otolaryngologic operations, with postoperative discomfort often lasting up to two weeks and affecting daily activities [5, 6]. Pain is most severe during swallowing, and inadequate control can delay recovery and compromise patient satisfaction. Opioid use in this setting is problematic, particularly for patients with OSA, who are at greater risk of respiratory depression. Consequently, multimodal analgesia has become the standard of care, incorporating acetaminophen, NSAIDs or COX-2 inhibitors, corticosteroids, and local anesthetic infiltration [7]. Despite these strategies, a substantial proportion of patients continue to experience significant pain, prompting investigation into adjuncts such as nefopam.

Nefopam is a centrally acting, non-opioid, non-NSAID analgesic with no respiratory depressive effects, making it an attractive option for OSA patients. It has demonstrated to improve pain control and lower opioid consumpution in several surgical settings, including abdominal, breast, and orthopedic procedures [8–16]. However, other studies—including those in spine, and thoracic surgery—have not demonstrated significant benefits [17–19]. This variability suggests that nefopam’s effectiveness may depend on the type of surgery, underlying pain mechanisms, and the extent of multimodal analgesia already provided.

Our results are consistent with those reporting no benefit. A key factor is the unexpectedly low morphine requirement in both groups. The median 24-hour consumption in our trial was only 5–7 mg, far lower than the 87 mg reported in Abdelmageed et al., which informed our sample size calculation [4]. The discrepancy may reflect differences in surgical techniques, patient populations, and, importantly, the consistent use of multimodal analgesia in our protocol. When baseline opioid needs are already low, any additional effect of nefopam may not be detectable.

Another possible explanation lies in the type of pain experienced after UPPP. The procedure produces intense somatic and inflammatory pain in the oropharynx, which may be well addressed by acetaminophen, NSAIDs, and local anesthetic infiltration. Nefopam may be more effective in settings where visceral or neuropathic pain predominates, as suggested by trials in cholecystectomy and spine surgery that reported benefits in reducing dysesthesia or visceral pain [14–16]. Pain perception may also vary by population; studies have suggested that Asian patients often report lower pain intensity than Western counterparts, which could further reduce the apparent benefit of adjunctive agents.

Although nefopam was generally well tolerated, we observed mild adverse events such as tachycardia and sweating, consistent with prior reports [8]. Importantly, no serious complications were noted, and the overall incidence of side effects did not differ significantly from the control group. The slow infusion regimen used in our protocol may have minimized adverse reactions compared with studies using more rapid administration.

This study has several limitations. The most important is the small sample size, which, combined with the unexpectedly low morphine requirements, left the trial underpowered to detect modest differences. Thus, our findings should be interpreted as exploratory. In addition, the study was conducted in a single center and included mainly young, relatively healthy adults, which may limit generalizability. We also did not assess long-term outcomes, such as chronic postoperative pain, where nefopam may still have a role.

Despite these limitations, our trial provides useful insights. To our knowledge, this is the first randomized controlled study of nefopam in UPPP. The consistently low morphine consumption across both groups underscores the effectiveness of multimodal analgesia in this setting and raises questions about the added value of adjuncts when baseline analgesia is already strong. Our findings suggest that nefopam should not be routinely recommended for UPPP, but they do not exclude the possibility that it could be beneficial in other surgeries characterized by higher opioid requirements or different pain mechanisms.

Future research should focus on identifying surgical contexts where nefopam offers the greatest benefit. Larger, multicenter trials are needed to confirm its role, ideally in procedures with higher postoperative opioid demands or where visceral or neuropathic pain is prominent. Comparative studies of dosing regimens, infusion rates, and routes of administration may also help optimize its use. Until then, multimodal analgesia with acetaminophen, NSAIDs, corticosteroids, and local anesthetic infiltration remains the mainstay for managing pain after UPPP.

## Conclusion

Nefopam did not significantly reduce 24-hour postoperative morphine consumption or improve pain scores, time to oral intake, or patient satisfaction after UPPP surgery. The incidence of adverse events was also comparable between nefopam and control group. Although our findings were insufficient due to the unexpectedly low opioid requirements, we do not recommend the use of nefopam in addition to standard multimodal analgesia for postoperative pain management in UPPP.

Abbreviations: IV PCA, intravenous patient-controlled analgesia; POD, postoperative day.
